# A comprehensive review on targeted therapies for triple negative breast cancer: an evidence-based treatment guideline

**DOI:** 10.1007/s12672-025-02227-6

**Published:** 2025-04-17

**Authors:** Hadir M. Emara, Nageh K. Allam, Rana A. Youness

**Affiliations:** 1 Nanotechnology Program, School of Sciences & Engineering, The American University in Cairo, New Cairo, 11835 Egypt; 2 Energy Materials Laboratory, Physics Department, School of Sciences & Engineering, The American University in Cairo, New Cairo, 11835 Egypt; 3 Department of Molecular Biology and Biochemistry, Faculty of Biotechnology, German International University, New Administrative Capital, Cairo, Egypt

**Keywords:** Triple negative breast cancer, Targeted therapy, Treatment guideline, Clinical trials

## Abstract

Triple-negative breast cancer (TNBC) is an aggressive malignancy characterized by limited therapeutic options and poor prognosis. Despite advancements in precision oncology, conventional chemotherapy remains the cornerstone of TNBC treatment, often accompanied by debilitating side effects and suboptimal outcomes. This review presents a comprehensive analysis of clinical trials on targeted therapies, aiming to establish a novel, evidence-based treatment strategy exclusively leveraging molecularly targeted agents. By integrating patient-specific genetic profiles with therapeutic responses observed across various clinical trial phases, this approach seeks to optimize efficacy while minimizing toxicity. The proposed targeted therapy combinations hold significant potential to revolutionize TNBC treatment, offering a paradigm shift toward precision medicine and improved patient outcomes.

## Introduction

Breast cancer (BC) is the second most commonly diagnosed cancer; accounting for 11.6% of all new cancer cases worldwide in 2022. In females, BC ranked as the leading cause of cancer and cancer related mortality worldwide in 2022 with the highest incidence rates [1]. BC is classified according to its immunohistochemical (IHC) features into 4 different subtypes which are luminal A, luminal B, Human epidermal growth factor receptor-2 (HER-2), and triple-negative breast cancer (TNBC). This classification is done based on the presence or the absence of estrogen receptor (ER), progesterone receptor (PR), HER-2 receptor, and the expression of cellular proliferation marker (Ki-67) [2].

Luminal A is characterized by ER and/or PR positivity, with HER-2 negativity, and Ki-67 expression of less than 20%. Luminal B shows ER and/or PR positivity, HER-2 positive or negative expression and Ki-67 of more than 20%. The HER2 subtype is defined by HER2 positivity and ER and PR negativity [3]. TNBC, which accounts for approximately 10–15% of all BC cases, lacks expression of ER, PR, and HER2 receptors, limiting available treatment options. As a result, TNBC is associated with the worst survival rates, higher aggression, earlier relapse, and poorer prognosis compared to other BC subtypes [4–6].

TNBC has been further classified into six subtypes based on IHC features: luminal androgen receptor (LAR), basal-like type 1 (BL1), basal-like type 2 (BL2), mesenchymal (MS), immunomodulatory (IM), and mesenchymal stem-like (MSL) [7, 8]. Other studies suggest classifications into four subtypes: LAR, MS, basal-like immunosuppressed (BLIS), and basal-like immune-activated (BLIA), or LAR, IM, MSL, and BLIS [9, 10]. The most recent classification includes five subtypes: LAR, IM, MS, BLIS, and an uncertain subtype [11]. Among these, BL1 and BL2 are the most frequently diagnosed, with incidence rates of 50–70% and 20–30%, respectively [3].

Currently, the main treatments for TNBC include chemotherapy, surgery, and radiotherapy [12]. However, these treatments are often associated with severe side effects, high resistance rates, and frequent recurrence [13]. In contrast, precision medicine, which involves targeting and inhibiting overexpressed proteins crucial for carcinogenesis using small-molecule drugs or monoclonal antibodies, has recently been introduced in TNBC management [14]. Although targeted therapies offer high efficiency and safety, they are typically used in second-line treatment or higher, either as monotherapy or in combination with chemotherapy [15–19]. Unfortunately, the limited number of approved targeted therapies and the absence of approved combination therapies mean that patients often experience either weak effects due to resistance to individual targets or severe side effects from chemotherapy combinations [20].

As a result, combining different targeted therapies has emerged as a growing trend in clinical trials. In this review, we examine the latest targeted drugs in TNBC treatment across different clinical trial phases and compare their efficiency and safety to existing treatments. Additionally, for the first time, we explore the overlap between TNBC subtypes suggested by various IHC studies by correlating the IHC features of TNBC patients in clinical trials with their response to targeted therapies. Ultimately, we propose an evidence-based treatment guide for TNBC patients that could inform future clinical trials involving targeted drug combinations.

## Advancements in the TNBC treatment

### Most recent therapeutic regimens for TNBC

Currently, the treatment guidelines for metastatic TNBC (mTNBC) are, to some extent, considering the protein expression profiles of different subtypes. For metastatic PD-1/PD-L1-positive TNBC, the first-line treatment involves PD-L1 inhibitors in combination with chemotherapy. This includes Pembrolizumab; Keytruda^®^ combined with paclitaxel, nab-paclitaxel, or carboplatin-gemcitabine, or Atezolizumab combined with nab-paclitaxel, though the latter has been withdrawn from the U.S. market [21]. PARP enzyme inhibitors such as Olaparib^®^ or Talazoparib^®^ are used as a second line of treatment for mTNBC with positive BRCA gene mutations or deletions after the chemotherapy [22, 23]. Meanwhile, Sacituzumab govitecan, an anti-TROP-2; Trodelvy^®^, is used as a third line of treatment or higher for mTNBC who have received at least one prior treatment for mTNBC [24]. While targeted therapies are already in use for TNBC treatment, there is still a lack of combinatorial targeted therapies and limited targeted treatment options available [15].

Moving to targeted drugs currently available in the market, including both approved and unapproved treatments for mTNBC that target various proteins in different subtypes. Those target proteins in mTNBC include Programmed death protein 1 (PD1)/Programmed death-ligand 1 (PD-L1) transmembrane proteins, Poly ADP Ribose Polymerases (PARPs), Trophoblast Antigen-2 (TROP-2) surface glycoprotein, Androgen Receptor (AR), Mammalian target of rapamycin (mTOR) as well as growth factor receptors (GFRs) and Receptor Tyrosine Kinases (RTKs).

PARP inhibitors such as Olaparib^®^, Talazoparib^®^, Lynparza^®^, and Talzenna^®^ are used with BRCA mutated subtypes. Where, the Lynparza^®^, and Talzenna^®^ were FDA approved in 2018 for the treatment of Breast Cancer gene 1 (BRCA1) and Breast Cancer gene 2 (BRCA2) mutations. BL1 and BL2 subtypes, characterized by inefficient DNA repair system as a result of common BRCA mutations or deletions, are particularly sensitive to the PARP targeting drugs as well. In addition, the BL2 subtype is also likely to be sensitive to anti-growth factor therapies such as Gefitinib, Cetuximab, and Lapatinib, due to its overexpression of growth factor receptors but they are not yet approved for the treatment of TNBC patients [25].

PD-1 and PD-L1 inhibitors, such as Atezolizumab (Tecentriq^®^), a PD-L1 inhibitor, and Pembrolizumab (Keytruda^®^), a PD-1 inhibitor, have been approved by the FDA for the treatment of PD-1/PD-L1 overexpressing mTNBC. This includes the IM subtype, which is particularly sensitive to immune checkpoint inhibitors due to the hyper-activation of immune pathways in these subtypes [25, 26].

Moving to mToR inhibitors, which are suitable for use in the MS subtype being characterized by its overexpression of mTOR receptors, while RTK inhibitors such as Dasatinib should be suitable for MSL subtype. Both targeted drugs were approved for treatment of other cancers but not yet approved for mTNBC [25, 27]. Finally, AR blockers, such as Bicalutamide and Enzalutamide, are targeted drugs that can be used in androgen receptor overexpressing cancers like LAR subtype. Although these drugs have been approved for other cancers, they have not yet been approved for TNBC as well [25, 28].

### Molecular targets in clinical trials

#### RTK and non-RTKs targeting drugs

The activities of different receptor tyrosine kinases (RTKs) play crucial roles in oncogenesis and the development of multiple cancers, including TNBC [29]. The activation and processing of RTKs involve multiple intersecting pathways with numerous intermediates. In some TNBC cases, this includes amplification or mutation of the RTKs themselves, while in others, it involves amplification or mutation of the intermediate pathways. Consequently, these intersecting RTK pathways lead to the activation of alternative pathways, which are a primary cause of resistance to RTK inhibitor monotherapies [30]. RTKs, including Epidermal Growth Factor Receptors (EGFRs), Vascular Endothelial Growth Factor Receptor (VEGFRs), Platelet Derived Growth Factor receptor (PDGFR), Fibroblast Growth Factor Receptor (FGFR), and c-MET receptor, are commonly amplified in various cancers, including TNBC.

Upregulation of growth factor receptors has been primarily observed in BL-2, MS and MSL subtypes [25]. EGFR exhibits approximately 89% overexpression across all TNBC subtypes, with 70–78% amplification in basal-like subtypes [31]. Additionally, VEGFRs overexpression is found in around 30–60% in TNBC patients, while PDGFR is significantly upregulated in TNBC patients compared to other BC subtypes, correlating with advanced BC stage, poorer prognosis and phenotype aggression [32]. FGFR exists in three different subtypes (FGFR1, FGFR2, and FGFR3), with amplification rates of 7–9%, 4% and <1%, respectively, in TNBC patients [33–36]. Lastly, c-MET shows approximately 10% amplification in TNBC patients [37].

On the other hand, non-receptor tyrosine kinases (non-RTKs) include Mitogen-activated protein kinase (MAPK/MEK), Phosphoinositide-3-kinases (PIK3), Protein Kinase B (AKT), mTOR, and Proto-oncogene tyrosine-protein kinase (c-Src) [38, 39]. PIK3/AKT/mTOR axis has been found to be upregulated in TNBC, with PI3KCA mutations commonly activated in TNBC cells. These mutations can be either AKT independent mutations, associated with the alterations in the intermediate pathways, such as PTEN loss or AKT-dependent mutations [40, 41]. Similarly, c-Src kinase is generally upregulated in TNBC tissues, while MAPK/MEK was also upregulated in approximately 40% of the TNBC patients [42].

The inhibition of RTKs and non-RTKS has been explored in TNBC at various stages, including clinical trials. However, due to the activation of alternative pathways, individual inhibition of RTKs and their pathway intermediates failed to provide adequate responses. Specifically, Cetuximab (EGFR inhibitor), Dasatinib (EGFR and HER-2 dual inhibitor), and Tivatinib (c-MET inhibitor) each demonstrated very low overall response rates (ORR) of 6%, 4.7%, and 5%, respectively, with minimal or no stable disease (SD) in Phase II clinical trials [43–45]. However, multiple RTK inhibitors and combinations therapies have shown more promising results, achieving higher disease control rates (DCRs). For example, Lucitanib, a pan-inhibitor targeting FGFR1-3, VEGFR1-3, and PDGFRα and β, achieved an overall response rate (ORR) of 26% when used as monotherapy [46]. In addition, the combination therapy of Apatinib and Camrelizumab—VEGFR2 and PD-1 inhibitors—achieved an overall response rate (ORR) of 43.3%, with 20% stable disease (SD), resulting in a disease control rate (DCR) of 63.3% [47]. Finally, the combination of non-RTKs, Trametinib and GSK2141795, MAPK and AKT inhibitors, achieved a DCR of 35.4%, with a 5% ORR and 30% SD (NCT01964924), as shown in Table 1.

**Table 1:** RTKs and non-RTKs targeting drugs in the treatment of metastatic TNBC (mTNBC) patients across different phases of clinical trials, either as monotherapy or in combination therapy

Targeted protein	General expression in TNBC	TNBC patients subtype and other characteristics in clinical trials	Clinical trial administered drug	Drug MOA	Phase of development	Efficiency	Clinical trial number	References
EGFR	89% over expression in all TNBC subtypes 70–78% of basal-like TNBC are overexpressing it	31 patients; mainly basal like subtype TNBC patients	Cetuximab	mAb against EGFR	Phase II clinical trials	The ORR was around 6% (2 patients) on monotherapy	NCICA58223	[44]
EGFR, HER-2, Src-kinase receptor		In 43 patients with locally advanced or mTNBC female patients	Dasatinib	EGFR, HER-2, and Src kinase receptor inhibitors	Phase II clinical trials	The ORR was 4.7% with 2 patients with partial response 2 patients had a SD		[43]
c-MET	Approximately 10% amplification in the TNBC patients		Tivatinib	c-MET inhibitor	Phase II clinical trials	Well tolerated but without enough efficiency In 22 patients with mTNBC, with 1–3 prior regimens of chemotherapy for mBC The overall response 5% with 1 PR patient, and another 5% stable disease	NCT01575522	[45]
FGFR1-3, vasoendotheli-al growth factor receptor) VEGFR1-3, and PDGFRα	The Fibroblast Growth Factor Receptor (FGFR), has four different subtypes including FGFR1, FGFR2, and FGFR3 with different amplification rates; 7–9%, 4% and <1%, in TNBC patients For the VEGFR 1–3 over-expression was found to be around 30–60% in TNBC patients The PDGFR was proved significantly upregulated especially in the TNBC patients more than other breast cancers	In phase III clinical trials 109 mTNBC patients were involved in the 10 mg dosing regimen In phase III clinical trials 68 patients only had EGFR1 amplification	Lucitanib	FGFR1-3, VEGFR1-3, and PDGFRα and β inhibitors	Phase I, Phase II clinical trial, and phase III clinical trials	In 27 patients ORR was 26% (7 of 27) and A PFS for all patients was 4 months In phase III clinical trials for determining the dose, 10 mg dosing regimen patients’ achieved better OS of 15 month than the 15 mg dosing regimen	NCT01283945	[46]
VEGFR + (PD-1)		40 patients with advanced TNBC	Apatinib + Camrelizumab	VEGFR2 inhibitor + A programmed cell death 1 (PD-1) inhibitor	Phase II clinical trials	Camrelizumab was added to continuous doses or intermittent doses of Apatinib for 30 and 10 patients respectively Continuous doses vs. intermittent dosing cohort had an ORR of 43.3% (13 of 30) vs. no objective response, a SD of 20%. Vs. 40.0% and PFS of 3.7 m vs. 1.9 m respectively	NCT03394287	[47]
MEK (MAPK) + AKT	(MAPK/MEK) is upregulated in approximately 40% of the TNBC patients AKT mutations are sometimes associated with PI3KCA AKT dependent mutations	In 37 patients with mTNBC	Trametinib + GSK2141795	MEK (MAPK) inhibitor + AKT inhibitor	Phase II clinical trials	The ORR was 5.4%, while SD rate was around 30% from the two groups and a median OS around 10 months	NCT01964924	https://clinicaltrials.gov/study/NCT01964924?cond=Breast%20Neoplasms,%20Triple-Negative&intr=MEK%20inhibitor%20&rank=1&tab=results#publications
PIK3 inhibitors	PI3KCA mutations found activated in most of the TNBC cells	In 20 patients with mTNBC	Buparlisib	PIK3 inhibitor	Phase II clinical trials	0% ORR However, a SD was achieved in 17 patients with a median OS of 11.2 m, and a median PFS of 1.8 m	NCT01790932 NCT01629615	[48]
mTOR + PI3K inhibitors		6 patients with TNBC were included in this study	Temsirolimus + Neratinib	mToR + PI3K inhibitors	Phase 1/2 clinical trials	0% ORR and a median of 2 months PFS However, the results of this combination was more satisfactory with the HER-2 positive group	NCT01111825	https://clinicaltrials.gov/study/NCT01111825?term=Temsirolimus%20Plus%20Neratinib%20for%20Patients%20With%20Metastatic%20HER2-Amplified%20or%20Triple%20Negative%20Breast%20Cancer&rank=1&tab=results#results-overview
mTOR + Aurora A kinase		In 47 patients with TNBC	Sapanisertib (MLN0128, INK128, TAK-228) + Alisertib (MLN8237)	A potent and selective mTOR inhibitor + A selective Aurora A inhibitor	Phase I clinical trial	The combination proved 100% tolerability in its all different dosage combinations	NCT02719691	https://clinicaltrials.gov/study/NCT02719691?cond=Breast%20Neoplasms,%20Triple-Negative&aggFilters=results:with,status:com&page=6&rank=56&tab=results#outcome-measures

Cetuximab, Dasatinib, Tivatinib, and the combination of Temsirolimus + Neratinib demonstrated very low disease control rates (DCR), while Buparlisib and the combination of Trametinib + GSK2141795 only achieved stable disease (SD) in mTNBC patients, all in phase II clinical trials. In contrast, Lucitanib and the combination of Apatinib + Camrelizumab achieved higher overall response rates (ORR) around 30% in phase III and phase II clinical trials, respectively

*ORR* overall response rate, *DCR* disease control rate, *OS* overall survival, *PFS* progression free survival, *SD* stable disease, *PR* partial response, *CR* complete response, *mTNBC* metastatic triple negative breast cancer

In fact, evidence supporting the activation of alternative pathways was confirmed by clinical studies that investigated various RTK monotherapies. In one study involving Cetuximab, tumor biopsies from 16 patients were analyzed 1 week before treatment, revealing activation of the EGFR pathway in 13 patients. However, after 1 week of treatment, inhibition of the EGFR pathway was only observed in 5 patients [44]. Furthermore, in another study involving c-MET targeting with Tivatinib, half of the patients developed EGFR amplification after treatment, which was suggested as a possible explanation for the drug’s low efficacy. This crosstalk between different RTKs, including the interaction between EGFR and c-MET, has been observed in various cancers, including breast cancer. Another factor proposed by the researchers for the low efficacy with Tivatinib was that only 10 out of 22 patients initially had c-MET amplification, yet this still does not account for the 5% response rate observed [45].

It comes as no surprise that Buparlisib, a monotherapy inhibiting only PI3K, achieved stable disease in metastatic TNBC patients, but none of the patients had any partial of complete response to the treatment [48]. Nevertheless, natural compounds with well-established anti-tumor activity, such as curcumin, have been found to block the activation of multiple RTKs, downregulate RTK expression, reduce the synthesis of RTK ligands, and inhibit RTK signaling pathways, along with non-RTK inhibition all at once [49]. In conclusion, the use of pan-RTK inhibitors like Lucitanib or the combination of the VEGFR-2 inhibitor Apatinib with the PD-1 blocker Camrelizumab has produced very promising results in terms of overall response rate (ORR) and disease control rate (DCR). Future treatment combinations should consider incorporating pan-RTK inhibitors such as Lucitanib, VEGFR-2 inhibitors like Apatinib, and anti-cancer natural products such as curcumin alongside PD-1 blockade to further enhance DCR and progression-free survival (PFS) in patients.

#### PARPs and PARPs targeting drugs

PARPs (poly (ADP-ribose) polymerases) are a family of 17 enzymes involved in the cell’s response to genotoxic stress. The PARP-1 is one enzyme of PARPs family whose basal activity is low, but it become activated in the presence of DNA damage. Upon activation, PARP-1 contributes to the formation of DNA repair complexes that include DNA ligases, DNA polymerases, and transcription factors. In the context of TNBC, approximately 15–25% of cases involve mutations in the BRCA genes, which are often hereditary. These mutations are associated with higher tumor aggression but are also linked to a better response to chemotherapy [50]. When the BRCA-1 and BRCA-2 genes are mutated, they lead to homologous recombination deficiency (HRD) in DNA repair. However, cancer cells with these BRCA mutations adapt to the resulting genomic instability by overexpressing PARP enzymes. As a result, PARP inhibitors have proven to be highly effective in treating BRCA-mutated TNBC subtypes [51]. The genome instability caused by HRD in BRCA-mutated cells makes them selectively vulnerable to PARP enzyme inhibition, which is essential for the cancer cells’ adaptation to these deficiencies. Additionally, emerging evidence suggests that combining PARP inhibitors with other targeted therapies could enhance their effectiveness, reduce the development of resistance, and sensitize the cells to treatment [52].

BRCA mutations have primarily been observed through IHC analyses in the BL-1, BL-2, and IM subtypes [25]. A review of clinical trials conducted to date on TNBC patients using PARP inhibitors and their combinations confirms the previously discussed theories. For instance, monotherapies with PARP inhibitors, such as Olaparib, achieved stable disease (SD) in 55% of BRCA-mutated patients, whereas none of the BRCA wild-type patients experienced stable disease or an overall response rate (NCT00679783), as shown in Table 2. On the other hand, a combination of the PARP inhibitor Olaparib and the PD-1 inhibitor Pembrolizumab in one study successfully achieved a disease control rate (DCR) of 80% in BRCA-mutated patients, while only a DCR of 44% was observed in BRCA wild-type patients [53]. In another study using the same drug combination for TNBC patients, without considering the BRCA status, a disease control rate (DCR) of 41.8% was achieved (NCT02657889). Furthermore, the combination of the PARP inhibitor Olaparib and the PI3K inhibitor Alpelisib achieved a DCR of 77% [54].

**Table 2:** PARP-targeted drugs in the treatment of TNBC patients in various phases of clinical trials, either as monotherapies or in combination therapies

Targeted protein	General expression in TNBC	Patients TNBC subtype	Drugs available	Drug MOA	Phase of development	Efficiency	Clinical trial number	References
PARPs	The prevalence of BRCA mutations, including expected PARPs adaptational changes occur in around 15–25% of TNBC	5 patients with positive BRCA mutations and 16 with negative BRCA mutations	Olaparib (AZD2281)	PARP inhibitor	Phase II clinical trials	In 21 evaluable TNBC patients the ORR for Olaparib alone was 0% 55% SD compared to 0% in BRCA positive compared to BRCA negative group A PFS of 3.5 and 1 months in BRCA positive and BRCA negative respectively	NCT00679783	https://clinicaltrials.gov/study/NCT00679783?cond=Breast%20Neoplasms,%20Triple-Negative&intr=PARP%20inhibitor&aggFilters=status:com&rank=4&tab=results#publications
PARPs		In 21 patients with recurrent Ovarian, Fallopian Tube, Primary Peritoneal, or Triple-Negative Breast Cancer that cannot be removed by surgery or metastatic	Olaparib and Onalespib	PARP inhibitors	Phase I clinical trial	The combination produced 0% ORR The maximum tolerated dose was determined for both drugs and it had no DLT at most of doses combinations	NCT02898207	https://clinicaltrials.gov/study/NCT02898207?cond=Breast%20Neoplasms,%20Triple-Negative&intr=onalespib&rank=2
PARP + PI3K		17 patients with recurrent TNBC with an average three prior failed lines of chemotherapies were involved in the study They were TNBC patients with BRCA mutations	Alpelisib + Olaparib	PI3K + PARPs inhibitors	Phase I study	ORR of 18%, a 59% SD rate, and a median PFS of 7.4 months with the combination therapy	NCT01623349	[54]
PARP + PD-L1		In 45 TNBC patients with metastatic inoperable, locally advanced, and who received in the 1st or 2nd line treatment a minimum of 4 cycles of platinum-based chemotherapy	Olaparib and Durvalumab	PARP and PD-L1 inhibitors		Less than 1% ORR with Olaparib alone or combined with Durvalumab	NCT03167619	https://clinicaltrials.gov/study/NCT03167619?term=Phase%20II%20Multicenter%20Study%20of%20Durvalumab%20and%20Olaparib%20in%20Platinum%20tReated%20Advanced%20Triple%20Negative%20Breast%20Cancer%20(DORA)%20(DORA)&rank=1
PARP + PD-1		In phase II 55 patients with advanced or mTNBC	Niraparib + Pembrolizumab (MK-3475)	PARP + programmed cell death 1 (PD-1) inhibitor	Phase II clinical study	In phase II the DCR was 41.8% with median PFS of 2.5 months, and median OS of 9.4 months	NCT02657889	https://clinicaltrials.gov/study/NCT02657889?cond=Breast%20Neoplasms,%20Triple-Negative&intr=PARP%20inhibitor&aggFilters=status:com&rank=10
PARP + PD-1		In 15 evaluable patients with BRCA mutations And 27 evaluable patients with BRCA wild type	Niraparib and pembrolizumab	PARPs + PD-1 inhibitors	Phase I and phase II clinical trial	The ORR was 47% and a SD of 33% with a median PFS of 8.3 months However, in BRCA wild type patients ORR was 11%, the DCR was 33%, and the median PFS was 2.1 months	NCT02657889	[53]

The combination of Alpelisib + Olaparib and Niraparib + pembrolizumab achieved higher DCR of 77% and 80%, respectively in phase I and phase II clinical trials, respectively, in BRCA mutated patients. In contrast, Olaparib and the combination of Olaparib + Onalespib achieved only stable disease (SD) in TNBC patients, in phase II and phase I clinical trials, respectively, in both BRCA positive and BRCA negative TNBC patients. While, prior treatment of patients with Platinum chemotherapy resulted in Olaparib and Durvalumab combination demonstrating very low disease control rates (DCR) with no ORR

*ORR* overall response rate, *DCR* disease control rate, *OS* overall survival, *PFS* progression free survival, *SD* stable disease, *PR* partial response, *CR* complete response, *DLT* disease limiting toxicity, *mTNBC* metastatic triple negative breast cancer

Unfortunately, neither the combination of the PD-L1 inhibitor Durvalumab and the PARP inhibitor Olaparib nor the combination of the two PARP inhibitors Olaparib and Onalespib were able to achieve any stable disease or response (NCT02898207 & NCT03167619). The resistance to the combination of the PD-L1 inhibitor Durvalumab and the PARP inhibitor Olaparib likely arose due to the requirement of four cycles of platinum-based therapy as a condition for joining the study. This therapy induced BRCA mutation reversion, even in BRCA-positive patients, making them resistant not only to the PARP inhibitors but also to the combination therapy with the PD-L1 and PARP inhibitors (NCT03167619).

To sum up, the response to PARP inhibitors is largely dependent on the BRCA status, particularly in the case of PARPi monotherapy, where only BRCA-positive mutation patients show a response. However, combining PARP inhibitors with other targeted treatments, such as PI3K inhibitors and PD-1 inhibitors, has been shown to achieve a higher disease control rate (DCR) in BRCA-positive patients, as well as modest DCR in BRCA-negative patients.

#### Immuno-targeted drug options

Certain cancers overexpress PD-1/PD-L1, allowing them to evade the immune system by inhibiting T cell activity through checkpoint inhibition [55, 56]. This T-cell checkpoint inactivation occurs upon binding of the PD-1 receptor, on the surface of T cells, to the PD-L1 ligand overexpressed on the cancer cells, leading to downstream signaling that inhibits T-cells proliferation [57–60]. The PD-1 and PD-L1 inhibitors have been extensively studied in the treatment of many cancers including, TNBC [61]. Although the FDA fast-tracked approval of these inhibitors, they ultimately failed to achieve the desired outcomes in subsequent clinical trials [21].

A review of clinical trials involving TNBC patients treated with PD-1 or PD-L1 inhibitors, either as monotherapies or in combination with other immune-targeted drugs, showed a maximum Disease Control Rate (DCR) of approximately 30%. For instance, the combination of the PD-L1 inhibitor Pembrolizumab and the Indoleamine 2,3-dioxygenase (IDO1) inhibitor Epacadostat^®^ resulted in a DCR of 30.5%, compared to approximately 24% with Pembrolizumab alone, as shown in two different studies (NCT02178722) [8, 62, 63]. Additionally, combinations of the PD-L1 inhibitor Durvalumab and the Cytotoxic T-lymphocyte associated protein 4 (CTLA-4) inhibitor Tremelimumab yielded DCRs of 20% and 29%, respectively, in separate studies, higher than the 8.3% DCR with Tremelimumab monotherapy (NCT02527434).

Interestingly, the monotherapy of the PD-1 inhibitor Spartalizumab demonstrated a higher DCR of 20% compared to its combination with the MHC class II ligand lymphocyte activation gene-3 (LAG-3) inhibitor Ieramilimab [64, 65]. In contrast, combination therapies involving PD-1 or PD-L1 inhibitors with other immune-targeted agents—such as the CD-122 (IL-2) inhibitor Ipilimumab, the CTLA-4 inhibitor Bempegaldesleukin, the CD-38 inhibitor Daratumumab, or the pan-kinase inhibitor Cabozantinib—produced DCRs of 14.9%, 17.6%, 16.8%, and 24.5%, respectively. Notably, the combination with Cabozantinib caused serious side effects in 71% of cases (NCT02983045, NCT03316586, NCT03098550, NCT02708680), as shown in Table 3.

**Table 3:** Immuno-targeted drugs for the treatment of TNBC in different phases of clinical trials both as monotherapies and in combination with other immunomodulatory therapies

Targeted protein	Patients TNBC subtype	Drugs available	Drug MOA	Phase of development	Efficiency	Clinical trial number	References
PD-L1	In 84 mTNBC patients Some of the patients had positive expression of PD-L1 and others had negative expression of PD-L1	Pembrolizumab	Anti PD-L1 receptor	Phase III clinical trial (FDA-approved)	The ORR was 21.4% in the PD-L1 positively expressing group vs. 0% in PD-L1 negatively expressing group The DCR was 23.8% in the first group The PFS was 2 months in both groups and the median OS was 18 and 9 months respectively	NCT02447003	[62, 63]
PD-L1	Some of the patients had positive expression of PD-L1 and others had negative expression of PD-L1 Equal no of patients with mTNBC were randomized either to the Pembrolizumab monotherapy group or the standard chemotherapy group	Pembrolizumab (MK-3475) OR Chemotherapy (Capecitabine, Eribulin, Gemcitabine, or Vinorelbine)	Anti PD-L1 receptor OR Chemotherapy	Phase III clinical trial (FDA approved)	The ORR in the 96 patients with PD-L1 + ve expression in Pembrolizumab group was 17.7% compared to 9.2% in the chemotherapy group The SD in patients with PD-L1 + ve expression group was 19.8% compared to 17.3% in the chemotherapy group The median OS of the patients with PD-L1 positive was 1 month longer in the patients assigned to the chemotherapy group	NCT02555657	[66]
PD-L1 + Indoleamine 2,3-dioxygenase 1 (IDO1)	In 36 TNBC patients, who received at least one standard medication for mTNBC	Pembrolizumab and Epacadostat	Anti-PD-L1 Monoclonal Antibody and selective indoleamine 2,3-dioxygenase (IDO1) inhibitor	Phase II clinical trial	An ORR was 11.1% with one complete response, and 3 partial response Additionally, 7 patients achieved SD (19.4%), while the median of PFS was 2 month and the median OS was 5.16 months	NCT02178722	https://clinicaltrials.gov/study/NCT02178722?cond=Breast%20Neoplasms,%20Triple-Negative&intr=pembrolizumab&aggFilters=phase:2,results:with,status:com&rank=7&tab=results#publications
PD-L1	In 12 patients with TNBC	Tremelimumab	PD-L1 inhibitor	Phase II clinical trials	The ORR was achieved by 8.3% patients, and a SD was achieved by 16.6% of patients The median OS was 13 months, while the PFS was around 3.5 months	NCT02527434	https://clinicaltrials.gov/study/NCT02527434?cond=Breast%20Neoplasms,%20Triple-Negative&aggFilters=results:with,status:com&page=8&rank=72&tab=results
PD-L1 and Cytotoxic T-lymphocyte associated protein 4 (CTLA-4)	12 TNBC patients Out of 12 TNBC patients, the 5 patients who showed disease progression on monotherapy were upgraded to a combination therapy	Tremelimumab and Durvalumab (MEDI4736)	PD-L1 and CTLA-4 inhibitor	Phase II clinical trials	58.3% DCR with the Tremelimumab monotherapy A stable disease was achieved only in 1 patient (20%) in the combination therapy The median overall survival for the patients was around 33 months, while the progression free survival was around 1 month	NCT02527434	https://clinicaltrials.gov/study/NCT02527434?cond=Breast%20Neoplasms,%20Triple-Negative&aggFilters=results:with,status:com&page=8&rank=72&tab=results
PD-L1 and CTLA-4	14 TNBC patients	Tremelimumaband Durvalumab (MEDI4736)	PD-L1 and CTLA-4 inhibitor	Phase II clinical trials	The ORR was 29%	NCT02536794	https://clinicaltrials.gov/study/NCT02536794?cond=Neoplasms,%20Breast&intr=CTLA-4&aggFilters=results:with,status:com&rank=1&tab=results#outcome-measures
PD-1	In 40 patients with TNBC	Spartalizumab (PDR001)	PD-1 receptor blocker	Phase I and II clinical trials	A DCR of 20% with all 8 patients achieving a SD and none with a response, while the PFS was 1.12 months	NCT02404441	[64]
PD-1 and LAG-3	In 21 mTNBC patients	Spartalizumab + Ieramilimab (LAG525)	Anti-PD-1 receptor LAG-3 inhibitor	Phase I and II clinical trial	The ORR receiving the combination therapy at lower doses was 14.3%	NCT02460224	[65]
PD-1 and HDAC	40 mTNBC patients	Atezolizumab and Entinostat	PD-1 and HDAC inhibitors	Phase I and II clinical trial	The ORR was 12.5% compared to the 2.5% in Atezolizumab only group	NCT02708680	https://clinicaltrials.gov/study/NCT02708680?cond=Breast%20Neoplasms,%20Triple-Negative&intr=Atezolizumab&aggFilters=results:with,status:com&rank=5&tab=results
PD-1 and CD38	In 41 patients with TNBC	Nivolumab + Daratumumab	PD-1 inhibitor and binds to CD38, inducing antibody-dependent cellular cytotoxicity apoptosis via, complement-dependent cytotoxicity, inhibition of mitochondrial transfer or antibody-dependent cellular phagocytosis	Phase II clinical trials	The DCR was 24.5% The PFS was 1.22 months It is also important to note that serious adverse events occurred in 70.7% of patients	NCT03098550	https://clinicaltrials.gov/study/NCT03098550?cond=Breast%20Neoplasms,%20Triple-Negative&aggFilters=results:with,status:com&page=9&rank=86
PD-1 + CD122 (IL-2)	In 429 patients with advanced solid tumors including TNBC	Bempegaldesle-ukin (NKTR-214) and Nivolumab	A first-in-class, CD122-preferential IL-2 pathway agonist and PD-1 receptor blocker	Phase II clinical trials	The ORR was 14.9% achieved in 64 patients	NCT02983045	https://clinicaltrials.gov/study/NCT02983045?cond=Breast%20Neoplasms,%20Triple-Negative&aggFilters=results:with,status:com&page=10&rank=97&tab=results#publications
PD-1 + CD122-( IL-2) + CTLA-4	In an expansion part 4 to the combination of Bempeglade sleukin and Nivolumab 17 patients with advanced solid tumors including TNBC	NKTR-214 (Bempegaldesleukin) and Nivolumab and Ipilimumab	PD-1 blocker and a first-in-class, CD122-preferential IL-2 pathway agonist that provides sustained activation of the IL-2 and CTLA-4 agonist	Phase I and phase II clinical trials	The ORR was 17.6% to the combination therapy	NCT02983045	https://clinicaltrials.gov/study/NCT02983045?cond=Breast%20Neoplasms,%20Triple-Negative&aggFilters=results:with,status:com&page=10&rank=97&tab=results#outcome-measures
PD-1 and Kinase	In 18 patients with TNBC	Nivolumab + Cabozantinib	PD-1 inhibitor + a kinase inhibitor, which inhibits the tyrosine kinase activity of MET, VEGFR-1, -2 and -3, AXL, RET, ROS1, TYRO3, MER, KIT, TRKB, FLT-3, and TIE-2	Phase II clinical trial	The DCR was 16.7% A median progression free survival of 3.6 months	NCT03316586	https://clinicaltrials.gov/study/NCT03316586?cond=Breast%20Neoplasms,%20Triple-Negative&aggFilters=results:with,status:com&page=5&rank=45&tab=results#outcome-measures
A TNF-related apoptosis inducing ligand (TRAIL)	In 5 evaluable TNBC patients	Imipridone ONC201	A TNF-related apoptosis inducing ligand (TRAIL)	Phase II clinical trials	The DCR was 20% with only one having a stable disease The progression free survival was found 1.2 months	NCT03394027	https://clinicaltrials.gov/study/NCT03394027?cond=Breast%20Neoplasms,%20Triple-Negative&aggFilters=results:with,status:com&page=5&rank=44&tab=results#publications
Aurora-A kinase	In 13 patients with different neoplasm; such as mTNBC, head and neck neoplasms, breast neoplasms, small cell lung carcinoma	AK-01 (LY3295668)	A highly specific Aurora-A kinase inhibitor	Phase I and II clinical trial	The Maximum Tolerated Dose (MTD) was found 25 mg twice daily The ORR that was 0% in only one assessed patient	NCT03092934	https://clinicaltrials.gov/study/NCT03092934?cond=Breast%20Neoplasms,%20Triple-Negative&aggFilters=results:with,status:com&page=9&rank=85#participation-criteria
CTLA-4 and Bruton’s tyrosine kinase (BTK)	105 patients with refractory metastatic solid tumors NSCLC (adenocarcinoma and squamous-cell carcinoma), BC (TNBC and HER2-positive), and pancreatic cancer (adenocarcinoma)	Durvalumab (MEDI4736) and Ibrutinib	Anti-PD-L1 and a small molecule drug that inhibits B-cell proliferation and survival by irreversibly binding the protein Bruton’s tyrosine kinase (BTK)	Phase II Clinical trials	The ORR was achieved by only 2 patients	NCT02403271	https://clinicaltrials.gov/study/NCT02403271?cond=Breast%20Neoplasms,%20Triple-Negative&aggFilters=results:with,status:com&page=11&rank=108&tab=results#outcome-measures

Pembrolizumab is the only immuno-targeted drug that has reached phase III clinical trials and was FDA-approved for PD-1/PD-L1 overexpressing metastatic TNBC (mTNBC) as a first-line treatment in combination with chemotherapy. In two separate phase III clinical trials, the Disease Control Rates (DCRs) were 23.5% and 37.5% in PD-1/PD-L1 positive mTNBC patients. Tremelimumab monotherapy achieved approximately 24.9% and 58.3% DCR in two phase II clinical trials. Both Spartalizumab and Imipridone monotherapies each achieved a DCR of around 20%. The combination therapies showed a DCR: (1) Pembrolizumab + Epacadostat (30%), (2) Tremelimumab + Durvalumab (30.5%), (3) Bempegaldesleukin + Nivolumab (14.9% and 17.6%), (4) Nivolumab + Cabozantinib (16.7%), (5) Spartalizumab + Ieramilimab (14.3%), (6) Atezolizumab + Entinostat (12.5%), all in phase II clinical trials. Although, Nivolumab + Daratumumab had a DCR of 24.5%, severe toxicities occurred in more than 70% of the patients. On the other hand, AK-01 had a 0% ORR in phase II clinical trials

*ORR* overall response rate, *DCR* disease control rate, *OS* overall survival, *PFS* progression free survival, *SD* stable disease, *PR* partial response, *CR* complete response, *DLT* disease limiting toxicity, *mTNBC* metastatic triple negative breast cancer

Finally, the combination of the PD-1 inhibitor Atezolizumab with the HDAC inhibitor Entinostat achieved an overall response rate (ORR) of 12.5%, compared to 2.5% with Atezolizumab alone (NCT02708680). These results suggest that even for the immune-modulatory TNBC subtype, patients do not show high responses to immunomodulatory drugs, even in combination therapies. This highlights the need for combining immunotherapy with other protein-targeting drugs to improve treatment outcomes.

### AR targeted drug options

The overexpression of the androgen receptor (AR) in the LAR subtype of TNBC is highly variable, ranging from 6.6 to 75%. This variability suggests that a diverse proportion of TNBC patients could potentially benefit from AR blockade [67]. One study specifically treating AR-overexpressing TNBC patients with the androgen receptor blocker Enzalutamide achieved a DCR of approximately 33%, compared to 0% in patients without AR overexpression.

In a separate study, Enzalutamide was combined with the PI3KCA inhibitor Taselisib in TNBC patients, regardless of their AR status, resulting in a Clinical Benefit Rate (CBR) of 35.7%. In this randomized trial, 19 TNBC patients were split into two groups: the Enzalutamide-only group showed a 0% CBR, while the combination group had a 35.7% CBR (NCT02457910). This highlights the likely role of PI3KCA inhibition, rather than AR blockade, in driving the observed clinical benefit. Therefore, it appears that only LAR patients—those with AR overexpression—are likely to respond to AR blockade, and combining AR inhibition with PI3KCA inhibition may improve DCR in these patients. Similarly, in a study done on AR positive TNBC patients treated with Bicalutamide (another AR blocker), the DCR was 32.1%, further emphasizing the highly variable distribution of the LAR subtype among TNBC patients (NCT02457910) [68].

On the other hand, the CYP17A1 inhibitor Orteronel, which suppresses androgen production, achieved only a 10% DCR in AR-overexpressing TNBC patients (NCT01990209). While, in a combination therapy study involving the selective androgen receptor modulator (SARM) Enobosarm and the PD-L1 inhibitor Pembrolizumab, the DCR was 25% in the AR-positive TNBC subgroup (NCT02971761), as summarized in Table 4.

**Table 4:** AR targeted drugs in treatment of TNBC in different phases of clinical trials as monotherapy or in combination with other targeted drugs

Targeted protein	General expression in TNBC	Patients TNBC subtype	Drugs available	Drug MOA	Phase of development	Efficiency	Clinical trial number	References
CYP17A1	Its expression is ranging from 6.6 to 75%	In 21 patients with TNBC Patients were AR + ve	Orteronel	Nonsteroidal, selective inhibitor of CYP17A1 that suppresses androgen production	Phase II clinical trials	The DCR was 9.6% divided equally into 4.8% ORR and 4.8% SD The PFS was 2 months, while the OS was 10.2 months	NCT01990209	https://clinicaltrials.gov/study/NCT01990209?cond=Breast%20Neoplasms,%20Triple-Negative&aggFilters=results:with,status:com&page=10&rank=94&tab=results#publications
AR		In 28 patients with AR positive, Estrogen Receptor Negative, and Progesterone Receptor Negative	Bicalutamide	Non-steroidal anti-androgens, which selectively block the androgen receptor (AR)	Phase II clinical studies	A median PFS over 1 year was achieved by 6 patients A DCR of 32.1% with all patients having a stable disease over 6 months	NCT00468715	https://clinicaltrials.gov/study/NCT00468715?intr=Bicalutamide&aggFilters=results:with,status:com&cond=breast%20neoplasms&rank=1&tab=results#publications
AR		Out of 118 patients with TNBC only 78 were evaluable Patients were AR + ve	Enzalutamide	AR inhibitor	Phase II clinical trial	A DCR of 33.3% A median PFS of 3.3 month A median OS of 17.6 months were obtained with fatigue being the only grade 3 adverse event	NCT01889238	[68]
AR + PI3KCA		19 patients with TNBC were randomized to two groups; where 14 received Enzalutamide and Taselisib, and 5 received Enzalutamide only	Enzalutamide + Taselisib	AR and PI3KCA inhibitor	Phase Ib/II clinical trial	A DCR of 35.7% was obtained in the combination treatment group Vs. 0% in the Enzalutamide only group A PFS of 3.4 months was achieved in the 14 patients’ group	NCT02457910	https://clinicaltrials.gov/study/NCT02457910?cond=Breast%20Neoplasms,%20Triple-Negative&intr=Enzalutamide%20&rank=5&tab=results#outcome-measures
SARM + PD-L1		In 16 patients with mTNBC, patients were AR positive	Enobosarm and pembrolizumab	SARM and PD-L1 inhibitors	Phase II clinical studies	Complete response was 6.3%, the partial response was 6.3%, and the stable disease was 12.5% (DCR = 25%) The median of overall survival was 25.5 month	NCT02971761	https://clinicaltrials.gov/study/NCT02971761?cond=Breast%20Neoplasms,%20Triple-Negative&intr=Enobosarm%20and%20pembrolizumab&rank=1&tab=results#publications

The combination therapies (Enzalutamide + Taselisib) achieved 35.7%, in AR negative TNBC patients, while (Enobosarm + Pembrolizumab) achieved 25%, in AR negative TNBC patients, in phase II clinical trials. Enzalutamide, Bicalutamide, and Orteronel exhibited a DCR of 33.3%, 31.2%, and 20.2%, respectively, in AR positive TNBC, in phase II clinical trials as well

*ORR* overall response rate, *DCR* disease control rate, *OS* overall survival, *PFS* progression free survival, *SD* stable disease, *PR* partial response, *CR* complete response, *DLT* disease limiting toxicity, *mTNBC* metastatic triple negative breast cancer

From these findings, it can be concluded that AR positivity should be a key factor in determining the efficacy of AR blockade in TNBC. Additionally, combining AR inhibition with PI3KCA inhibition should be considered, especially for AR-positive TNBC patients, as this combination could potentially double the response rate in the LAR TNBC subtype [25]. Moreover, the combination of SARMs with PD-L1 inhibitors may be particularly beneficial in immunomodulatory, AR-positive LAR TNBC patients.

### Other targeted drug options

The HSP-90 inhibitor Ganetespib has demonstrated its potential for treating metastatic cancers across all subtypes. In a clinical trial, it achieved a 100% Overall Response Rate (ORR) in 20 patients with metastatic breast cancer who had received at least one and up to three prior treatments. However, 27.27% of the patients experienced serious adverse events (NCT01273896). Additionally, the Exportin-1 inhibitor Selinexor achieved a DCR of 30% in TNBC patients [69]. Moreover, the combination between the HDAC inhibitor Panobinostat and the aromatase inhibitor Letrozole achieved a DCR of 58.3% in TNBC patients [70].

Furthermore, the technique of targeted drug delivery using upregulated cellular receptors to selectively deliver chemotherapeutic agents inside cancer cells has shown success. For example, the TROP-2 monoclonal antibody (mAb) Sacituzumab Govitecan effectively directed the targeted delivery of the topoisomerase inhibitor Irinotecan, achieving a Disease Control Rate (DCR) of 45.4% [24]. Additionally, the folate receptor monoclonal antibody (mAb) Mirvetuximab enabled targeted delivery of the microtubule inhibitor Soravtansine, achieving a 50% Disease Control Rate (DCR). However, the limitation of the Mirvetuximab-Soravtansine clinical trial was the small sample size, with only two patients involved. Future studies should include a larger cohort of TNBC patients to better assess its efficacy (NCT03106077).

Another treatment option, relaying on targeted drug delivery using upregulated cellular receptors, is Cofetuzumab Pelidotin, an antibody–drug conjugate (ADC) consisting of a monoclonal antibody (mAb) targeting the upregulated protein tyrosine kinase 7 (PTK7). This mAb is linked to Auristatin-0101, a potent microtubule inhibitor. In a clinical trial involving 29 TNBC patients, this treatment achieved a DCR of around 50%, with all clinical data summarized in Table 5. The mechanism of action of PTK7 targeting involves inhibiting the activation of the mitogen-activated protein kinase kinase-7 (MKK7), which in turn blocks the activation of c-Jun N-terminal kinase (JNK). This cascade ultimately inhibits the activation of the activator protein-1 (AP-1) complex, a key regulator in cellular processes [71].

**Table 5:** Different potential targeted drugs for the treatment of TNBC in the clinical trials as monotherapy or in combination therapy

Targeted protein	Patients TNBC subtype	Drugs available	Drug MOA	Phase of development	Efficiency	Clinical trial number	References
Hsp90	In 20 mBC patients including mTNBC	Ganetespib	Hsp90 inhibitor	Phase II clinical studies	The overall response rate was 100%	NCT01273896	https://clinicaltrials.gov/study/NCT01273896?cond=Breast%20Neoplasms&intr=ganetespib&rank=2&limit=10&aggFilters=status:com&tab=results#outcome-measures
Aromatase and HDAC	In 12 mTNBC patients	Letrozole and Panobinostat	Aromatase inhibitor; that convert androgens into estrogen and HDAC inhibitors	Phase I clinical trials	The partial response rate was ~ 16.7% the SD rate ~ 41.6% However, the Thrombocytopenia was the most common severe adverse event with a rate of 33.3%	NCT01105312	[70]
TROP-2	In 108 mTNBC patients with at least two prior failed anticancer therapies	Sacituzumab-Govitecan	mAb against TROP-2, hRS7, that selectively deliver its covalently linked SN-38 (irinotecan) through linker; a topoisomerase I inhibitor	Phase II clinical trials/FDA approved	The ORR was 33% in monotherapy, with 3 complete response and 30 partial response The DCR was 45.4%	NCT01363232	[24]
TROP-2	In 468 patients with mTNBC but without brain metastases with All patients treated with Taxanes in a prior line of treatment	Sacituzumab-Govitecan OR chemotherapy of choice (gemcitabine, vinorelbine, capecitabine, or eribulin)	mAb against TROP-2, hRS7, that selectively deliver its covalently linked SN-38 (irinotecan) through linker; a topoisomerase I inhibitor	Phase III clinical trial	The ORR was 35% with Sacituzumab-Govitecan compared to 5% only with chemotherapy The median OS was 12.1 vs. 6.7 months in Sacituzumab-Govitecan vs. chemotherapy group respectively	NCT02574455	
Exportin-1	In 10 patients with TNBC	Selinexor (KPT-330)	A first-in-class selective inhibitor of nuclear export which, through inhibition of exportin-1, causes accumulation of tumor suppressor proteins, reduction in oncoproteins, and apoptosis of plasma cells	Phase I and Phase II clinical trials	Selinexor showed a good tolerability in patients The DCR was 30%	NCT02402764	[69]
Folate receptor	Only 2 TNBC patients were evaluable	Mirvetuximab Soravtansine	A folate receptor alpha directed antibody and microtubule inhibitor conjugate	Phase II clinical trials	1 patient had achieved SD the other one had a progressive disease Contradictory to the radiographic response rate was 100%	NCT03106077	https://clinicaltrials.gov/study/NCT03106077?cond=Breast%20Neoplasms,%20Triple-Negative&aggFilters=results:with,status:com&page=8&rank=76
Protein tyrosine kinase 7 (PTK7)	In 29 patients with TNBC	Cofetuzumab Pelidotin (PF-06647020)	Antibody–drug conjugate (ADC) for the mAb against protein tyrosine kinase 7 (PTK7), which specifically deliver Monomethyl auristatin E, potent inhibitor of tubulin assembly	Phase I clinical trials	The DCR was 48.3% with a PFS of 1.5 month with 6 patients had achieved partial response to the treatment and 8 patients had a SD	NCT02222922	[71]
CD-71	In 45 adults with advanced solid tumors including TNBC	CX-2029 monomethyl auristatin E (MMAE) conjugated to the cytotoxin	Drug Conjugate of 1-Cytotoxin Targeting CD71 (Transferrin Receptor) 2-Monomethyl auristatin E (MMAE), anti-tubulin polymerization, via a cysteine protease-cleavable dipeptide linker	Phase I clinical trials	No disease limiting toxicities (DLT) were observed at 4 mg/kg dose The phase II recommended dose was determined to be 3 mg/kg	NCT03543813	[72]

Sacituzumab-Govitecan exhibited ORR of 35% in phase III clinical trials, regardless of TNBC subtype. It has been approved as a third line treatment in mTNBC, with at least one prior treatment for mTNBC. Cofetuzumab Pelidotin and Selinexor achieved DCRs of 48.3% and 30%, respectively, without considering TNBC subtypes, in phase II clinical trials. Ganetespib had a 100% ORR, but with severe toxicities occurring in 27.27% of the patients. The combination (Letrozole + Panobinostat) achieved 58.3% DCR, but Thrombocytopenia was a DLT in 33.3% of the patients. Mirvetuximab Soravtansine achieved 50% DCR, but in only two patients

*ORR* overall response rate, *DCR* disease control rate, *OS* overall survival, *PFS* progression free survival, *SD* stable disease, *PR* partial response, *CR* complete response, *DLT* disease limiting toxicity, *mTNBC* metastatic triple negative breast cancer

## Discussion

Since 2013, an immunohistochemical (IHC) study of 83 TNBC patient samples by Mrkli I. et al. identified overlapping characteristics between the BL subtype and LAR subtype, the BL subtype and IM subtype, as well as between the BL subtype, IM subtype, and LAR subtype [73]. Moreover, the literature indicates that non-receptor tyrosine kinase (non-RTK) PI3KCA mutations are prevalent across nearly all TNBC subtypes, while BRCA mutations and receptor tyrosine kinase (RTK) mutations are particularly common in the BL1 subtype, as well as BL2 subtypes [25, 41]. By correlating the results of the reviewed clinical trials with TNBC patient subtypes, IHC data, and the targeted therapy regimens used, this study demonstrates the heterogeneous and overlapping nature of TNBC subtypes.

Concerns about the need for targeted combination therapy was raiesd by a phase II clinical trial involving 193 TNBC patients, who were either randomized or assigned to Arm A or Arm B. Patients in Arm B, who received the physician’s chemotherapeutic drug of choice, showed overall response (OR) and disease-free survival (DFS) rates similar to those of patients in Arm A, who received genomic-guided monotherapies [74]. In the light of this, it can be concluded that relying on the genomic directed targeted monotherapies, same efficiencies of the chemotherapeutics can be obtained but with much lower side effects. Hence, those side effects can be much decreased while efficacies can be much improved using the targeted combination therapies.

To illustrate the key clinical trials reviewed in our study, we present the correlation between targeted therapy regimens, patient characteristics, and the Disease DCRs achieved. This highlights the significance of each clinical trial in developing the evidence-based guideline for TNBC suggested. For instance, the combination of the PARP inhibitor Olaparib with the PI3K inhibitor Alpelisib showed enhanced efficacy, particularly in highly resistant and recurrent metastatic TNBC (mTNBC) BL1 and BL2 subtypes with known BRCA mutations. This combination achieved a DCR of 77% (18% Overall Response Rate [ORR] and 59% Stable Disease [SD]), compared to a 55% stable disease rate in BRCA-positive TNBC patients treated with Olaparib monotherapy [48, 54]. This highlights the common mutations in the PI3K and BRCA genes found in the BL1 and BL2 subtypes, underscoring the importance of combination therapies using PARP inhibitors and PI3K inhibitors in BRCA-positive BLIA and BLIS subtypes. Secondly, the combination of the VEGFR-2 inhibitor Apatinib and the PD-1 inhibitor Camrelizumab achieved a DCR of 63.3%, compared to a DCR of only 20% stable disease (SD) with the PD-1 inhibitor Spartalizumab monotherapy [47, 64]. This highlights the potential for RTK upregulation in the BLIA or IM subtypes.

Additionally, the combination of PARP inhibitor Olaparib with PD-1 inhibitor Pembrolizumab achieved an 80% Disease Control Rate (DCR) in BRCA-mutated BL1 and BL2 subtypes, compared to 55% stable disease (SD) with Olaparib monotherapy in BRCA-positive patients. On the other hand, the maximum DCR with immunotherapy alone was only 30%, even in PD-1/PD-L1-positive TNBC patients, as shown in Table 3 (NCT02657889) [54]. This confirms the overlapping nature between BL1, which overexpresses RTKs such as VEGFR-2, and BL2, which overexpresses non-RTKs such as PI3K, with the IM subtype, as represented by the newer classifications of TNBC into BLIS and BLIA subtypes.

The PI3K overexpression in most TNBC patients was also validated in a clinical trial using the PI3K inhibitor Buparlisib, where 85% stable disease (SD) for more than 11 months was achieved with monotherapy, without determining the TNBC subtype [48]. The PI3K inhibitor Taselisib and the AR blocker Enzalutamide resulted in a 35.7% DCR in TNBC patients, regardless of their subtype. However, this DCR was most likely attributed to the PI3K inhibitor Taselisib, as the patients’ negative AR expression was suggested by the 0% response to Enzalutamide monotherapy in the same study (NCT02457910) as shown in Table 4. This re-confirms the efficacy of PI3K inhibitors in most of TNBC subtypes including mTNBC patients including AR subtype. In an IHC study by Yin et al., mTOR was found to be upregulated in the MS, MSL, and LAR subtypes [25]. To confirm this, a clinical trial involving mTOR inhibition in MS TNBC patients achieved a 40% DCR with high rates of complete and partial responses when Everolimus was combined with Bevacizumab and Doxorubicin [25, 75]. This proves the upregulation of growth factor such as VEGF-A and non-RTK such as mToR in MS TNBC. However, substituting the chemotherapeutic agent with a PIK3CA inhibitor, based on literature supporting its specific upregulation in MS, MSL, and AR TNBC subtypes, could further improve both efficacy and safety.

In a phase III study, the superior efficacy of anti-TROP2 Sacituzumab-Govitecan was demonstrated, with an ORR of 35% on monotherapy compared to just 5% efficacy with chemotherapeutic monotherapy in resistant mTNBC patients [24]. In another study, Sacituzumab-Govitecan achieved a 45.5% DCR on monotherapy in 108 mTNBC patients who had at least two prior failed anticancer therapies. It is important to note this clinical trial done without considering the subtyping of mTNBC patients, where HSP-90 inhibitor achieved a 100% response rate, though it caused severe side effects in around 30% of the patients (NCT01273896), as shown in Table 5. This highlights its potential for future combination with other targeted therapies, aiming to lower the dose while enhancing both safety and efficacy.

Consequently, due to the heterogeneous yet overlapping nature of TNBC, the adoption of a three-drug combination therapy regimen should be further emphasized. This approach has already demonstrated effectiveness in TNBC treatment, as shown in a clinical trial where the combination of liposomal Doxorubicin, Bevacizumab, and Everolimus achieved a 40% DCR in 39 patients, with 4 complete responses and 7 partial responses. Despite being a combination of one chemotherapeutic and two targeted therapies, this regimen resulted in a notably high response rate [75]. Unfortunately, a combination of three targeted therapies has not yet been explored, particularly in clinical trials. However, the positive results from two-targeted therapy combinations, coupled with the overlapping nature of TNBC subtypes, underscore the need for future clinical trials investigating three-targeted therapy combinations (Table 6).

**Table 6:** A comparison between the guideline’s standard treatment, and the recommended evidence based guide for the BRCA mutated BL-1 and BL-2 with or without AR subtype nature

BRCA mutated BL1 and BL2 with or without AR subtype nature	Guidelines’ standard treatment	Recommended evidence based guide	Recommended future clinical trials
Line of treatment	*1st line of treatment*: Chemotherapy; Paclitaxel, or nab-paclitaxel, or Carboplatin-Gemcitabine [76]	*1st line of treatment*: PARP-I (Olaparib) + PI3K_i_ (Alpelisib) [54]	PARP_i_ (Olaparib) + PI3K_i_ (Alpelisib) + AR blocker (Enzalutamide)/or VEGFRi (Apatinib/Lucitanib)
	*2nd line of treatment*: PARPs monotherapy; Olaparib and Talazoparib [22]	*2nd line of treatment*: PARP-I (Olaparib) + PI3K_i_ (Alpelisib) + anti-TROP-2 [24, 54]	
	*3rd line of treatment*: The anti-TROP-2, or Sacituzumab govitecan with at least one prior treatment for the metastatic TNBC [24]	*3rd line of treatment*: HSP-90 (Geldanamycin) inhibitor either as monotherapy (NCT01273896)	
ORR/PCR	*1st line of treatment*: In one clinical trial, patients were divided into three groups; group 1, 2 and 3 with group1 (Nab-Paclitaxel + Carboplatin), (Nab-Paclitaxel + Gemcitabine), and (Gemcitabine + Carboplatin) respectively The ORR were 79.6%, 43.8%, and 29.2% for group 1, 2, and 3 respectively NCT01881230 [76]	*1st line of treatment*: ORR = 18%	NA In the two-drug targeted combination therapy, a DCR of 77% was obtained vs. 79.6% in the best achieving first line combination chemotherapy. However, the severe and serious side effects including the grade 3 or higher adverse events and the serious side effects in the chemotherapy groups were incomparable to any other treatment. Hence, the three-drug targeted combination therapy is expected to even enhance the overall response rate with a much greater patient safety and reliability The AR blocker is advised to be the third drug in case of AR positive and BRCA mutated BL1 and BL2, whereas the VEGFR inhibitor such as Apatinib or Lucitanib is advised to be used as a third drug in case of BRCA mutated BL1 and BL2 without AR nature [25]
	*2nd line of treatment*: In one study, carried on TNBC patients with BRCA mutations, the ORR to Olaparib was 0% [73] NCT00679783	*2nd line of treatment*: NA	
	*3rd line of treatment*: 35% with sacituzumab govitecan 5% with chemotherapy [24]	*3rd line of treatment*: HSP-90 inhibitor produced 100% ORR in mTNBC regardless of their subtyping But with severe side effects in around 30% of the patients	
DCR	*1st line of treatment*: The DCR were 79.6%, 43.8%, and 29.2% for group 1, 2 and 3 respectively	*1st line of treatment*: DCR = 77%	NA
	*2nd line of treatment*: DCR = 55% with Olaparib monotherapy	*2nd line of treatment*: NA	
	*3rd line of treatment*: DCR = 35% with sacituzumab govitecan	*3rd line of treatment*: DCR = 100%	
Others (PFS/OS)	*1st line of treatment*: PFS = 5.5, 8.3, and 6 months OS = 12.1, 16.8, and 12.6 months in Groups 1, 2, and 3, respectively	1st line of treatment: PFS = 7.4 months	NA
	*2nd line of treatment*: OS = 19.3 months		
	*3rd line of treatment*: PFS = 5.6 months vs. 1.7 months with chemotherapy OS = 12.1 months vs. 6.7 months with chemotherapy		
The grade 3 or 4 side effects and the dose limiting toxicities (DLT)	*1st line of treatment*: Treatment-related grade 3 or higher side effects affected 58.3%, 68.25%, 71.89% in Groups 1, 2, and 3, respectively. Additionally, serious adverse events affected 31.25% of the patients in group 2, which included febrile neutropenia, myocardial infarction, pneumonia, femur fracture, metastases to central nervous system, metastases to meninges, depressed level of consciousness, mental status changes, pulmonary embolism, and deep vein thrombosis [76]	*1st line of treatment*: The combination therapy was generally tolerated in BRCA negative and BRCA positive TNBC population The treatment-related grade 3 or 4 adverse events were hyperglycemia in 18% and rash in 12%. No treatment-related deaths occurred. The dose-limiting toxicities of hyperglycemia and febrile neutropenia occurred in two patients	NA
	*2nd line of treatment*: Nausea, vomiting, and decreased appetite were the adverse events [78]		
	*3rd line of treatment*: The adverse events of grade 3 or higher were: 1. Neutropenia (51% with sacituzumab govitecan and 33% with chemotherapy) 2. Leukopenia 3. Febrile neutropenia 4. Although, there were three deaths in each group; no deaths occurred as a result of Sacituzumab-govitecan treatment [24]		
Patients Characteristics	*1st line of treatment*: TNBC adenocarcinoma patients with one prior anthracycline line of treatment or no prior lines of treatment	*1st line of treatment*: Advanced recurrent metastatic TNBC with an average of 3 lines of chemotherapy for metastatic disease. The TNBC were of BL1 and BL2 with BRCA mutations	NA It should be also noted that the patient characteristics in the chemotherapy group were much better and earlier stages than those in the clinical trials of the targeted combination therapy who were with advanced, metastatic, and recurrent TNBC
	*2nd line of treatment*: The study was done on TNBC with BRCA mutations as well		
	*3rd line of treatment*: Confirmed TNBC, relapsed after at least two prior standard therapeutic regimens for advanced/metastatic TNBC. All the TNBC patients included had failed Taxane therapy		
Price and feasibility	*1st line of treatment*: Paclitaxel Trade name: Paclitaxel^®^ Abraxane^®^ Price: ranges from $14/injection to $1659/injection https://www.drugs.com/price-guide/paclitaxel https://www.drugs.com/price-guide/abraxane	*1st line of treatment*: Olaparib Trade name: Lynparza^®^ Price: $9071/60 tablets https://www.drugs.com/price-guide/lynparza + Alpelisib Trade name: Piqray^®^ Price: $24,859/56 tablets https://www.drugs.com/price-guide/piqray	Olaparib Trade name: Lynparza^®^ Price: $9071/60 tablets https://www.drugs.com/price-guide/lynparza + Alpelisib Trade name: Piqray^®^ Price: $24,859/56 tablets https://www.drugs.com/price-guide/piqray + Enzalutamide Trade name: Xtandi^®^ Price: $15,571/120 capsules https://www.drugs.com/price-guide/tukysa OR + Lucitanib Trade name: Tukysa^®^ Price: $13,789/60 tablets https://www.drugs.com/price-guide/tukysa Apatinib N/A
	*2nd line of treatment*: Olaparib Trade name: Lynparza^®^ Price: $9071/60 tablets https://www.drugs.com/price-guide/lynparza or Talazoparib Trade name: Talzenna^®^ Price: $19,784/30 tablets https://www.drugs.com/price-guide/talzenna	*2nd line of treatment*: Olaparib Trade name: Lynparza^®^ Price: $9071/60 tablets https://www.drugs.com/price-guide/lynparza + Alpelisib Trade name: Piqray^®^ Price: $24,859/56 tablets https://www.drugs.com/price-guide/piqray + Sacituzumab govitecan Trade name: Trodelvy^®^ Price: $2633.07/one injection https://www.drugs.com/price-guide/trodelvy	
	*3rd line of treatment*: Sacituzumab govitecan Trade name: Trodelvy^®^ Price: $2633.07/one injection https://www.drugs.com/price-guide/trodelvy	*3rd line of treatment*: Geldanamycin N/A	

*ORR* overall response rate, *DCR* disease control rate, *OS* overall survival, *PFS* progression free survival, *SD* stable disease, *PR* partial response, *CR* complete response, *DLT* disease limiting toxicity, *mTNBC* metastatic triple negative breast cancer

Consequently, to develop an updated evidence-based treatment guideline, we conducted, to the best of our knowledge for the first time, a comparison between the outcomes of the most recently approved treatment guidelines and those of the proposed treatment guidelines across different TNBC subtypes. Additionally, we present future recommendations for drug combinations that are not yet explored in current clinical trials.

Starting with the BL-1 and BL-2 TNBC subtypes, which commonly carry BRCA mutations, the first-line treatment recommended by the guidelines showed a DCR of 79.6% in the highest achieving combination therapy of Nab-Paclitaxel and Carboplatin, although it caused severe, life-threatening side effects [76]. However, our suggested alternative for this group as a first-line therapy involves a PARP inhibitor and PI3K inhibitor, which achieved a 77% DCR with almost no or mild side effects, even when considering the more severe clinical presentation of the TNBC patients in this study compared to the previous one [54]. Furthermore, when considering the second-line Olaparib monotherapy, which resulted in only a 55% DCR in clinical trials, we recommend adding a PI3K inhibitor to the PARP inhibitor and the anti-TROP2 agent, Sacituzumab govitecan. This recommendation is based on the synergistic effect of combining PARP and PI3K inhibitors, along with the high efficacy of Sacituzumab govitecan, even in resistant mTNBC patients [54, 75]. While, the third line therapy may include HSP-90 inhibitor in combination with one or more of the prior line treatments based on the patients’ response to them (NCT01273896).

Moving to the comparison between the standard and the recommended treatments for IM TNBC subtype with or without BRCA mutations. The first line standard treatment includes Pembrolizumab and chemotherapy; Paclitaxel, or nab-paclitaxel, or Carboplatin-Gemcitabine, which achieved 65% DCR in the clinical trials compared to 80% obtained with VEGFR2-inhbitor; Apatinib and the PD-1inhibitor; Camrelizumab with extensively lower side effects and no DLT in the later and taking into consideration the better clinical picture of TNBC in the former one [47, 77]. Regarding our recommendations for later line of treatments and future clinical trials, the addition of, PARP inhibitor such as Olaparib in the co-positive PD-1/PD-L1 and BRCA or PI3Ki; Alpelisib in the BRCA negative IM subtype, to the prior treatment line of VEGFR2-I; Apatinib and the PD-1 inhibitor; Camrelizumab as detailed in Table 7.

**Table 7:** A comparison between the guideline’s treatment, and the recommended evidence based guide for the IM subtype nature with or without BRCA mutations

IM subtype with or without BRCA mutated BL1 and BL2 subtype nature	Guidelines’ treatment	Recommended evidence based guide	Recommended future clinical trials
Lines of treatment	The standard 1st line of treatment includes a combination between Pembrolizumab and chemotherapy; Paclitaxel, or nab-paclitaxel, or Carboplatin-Gemcitabine [77]	VEGFR2 i (Lucitanib) + PD-1 I (Camrelizumab) [47]	VEGFR2 i (Apatinib) + PD-1 i (Camrelizumab) + PARP_i_ (Olaparib) or PI3Ki (Alpelisib)
ORR/PCR	ORR = 52.7%	ORR = 47%	NA
DCR	DCR = 65%	DCR = 80%	NA
Others (PFS/OS)	PFS = 9.7 months OS = 23 months	PFS = 2.5 months OS = 9.4 months	NA
The disease limiting toxicities (DLT)	DLT = 30% Severe adverse events included febrile neutropenia, thrombocytopenia, MI, cardiac arrest, cardiac failure, cardiorespiratory arrest, adrenal insufficiency, intestinal obstruction, gastric ulcer hemorrhage, lower gastrointestinal hemorrhage, pancreatitis, hepatitis, autoimmune hepatitis, around 10% infections, limb injury, lumbar vertebral injury, and death	In the whole population, the grade three or higher adverse events were as follow: 1. Anemia [18%] 2. Thrombocytopenia [15%] 3. Fatigue [7%] 4. Immune-related adverse events [4%]	NA In the two-drug targeted combination therapy, a DCR of 80% was obtained vs. 65% in first line combination with chemotherapy. However, the severe and serious side effects and the serious DLT in the chemotherapy groups were incomparable to any other treatment. Hence, the three-drug targeted combination therapy is expected to even enhance the overall response rate with a much greater patient safety and reliability The PARP inhibitor is advised to be the third drug in case of BRCA mutated, IM subtype, whereas the PI3K inhibitor such as Alpelisib is advised to be used as a third drug in case of IM only nature
Patients characteristics	TNBC patients with locally recurrent or metastatic breast cancer that was not previously treated with chemotherapy	Patients with advanced or metastatic TNBC with at least 3 chemotherapeutic lines for metastatic TNBC	NA
Price and feasibility	Pembrolizumab Trade name: Keytruda^®^ Price: $6046/one injection https://www.drugs.com/price-guide/keytruda + Paclitaxel Trade name: Paclitaxel^®^ Abraxane^®^ Price: ranges from $14/injection to $1659/injection https://www.drugs.com/price-guide/paclitaxel https://www.drugs.com/price-guide/abraxane	Pembrolizumab Trade name: Keytruda^®^ Price: $6046/one injection https://www.drugs.com/price-guide/keytruda + Lucitanib Trade name: Tukysa^®^ Price: $13,789/60 tablets https://www.drugs.com/price-guide/tukysa	Pembrolizumab Trade name: Keytruda^®^ Price: $6046/one injection https://www.drugs.com/price-guide/keytruda + Lucitanib Trade name: Tukysa^®^ Price: $13,789/60 tablets https://www.drugs.com/price-guide/tukysa + Olaparib Trade name: Lynparza^®^ Price: $9071/60 tablets https://www.drugs.com/price-guide/lynparza

*ORR* overall response rate, *DCR* disease control rate, *OS* overall survival, *PFS* progression free survival, *SD* stable disease, *PR* partial response, *CR* complete response, *DLT* disease limiting toxicity, *mTNBC* metastatic triple negative breast cancer

Unfortunately, in the MS, MLS and the AR TNBC subtypes, there are no available standard treatments. But, based on our review, there are recommended combinations in Table 7 and Table 8 for future clinical trials and others with proven efficacy and safety from different levels of clinical trials in the treatment of TNBC with those subtypes.

**Table 8:** A comparison between the guideline’s treatment, and the recommended evidence based guide for the MS and MSL subtypes

MS and MSL subtypes	Guidelines’ treatment	Recommended evidence based guide	Recommended future clinical trials
Line of treatment	No standard treatments are available for the MS and MSL subtypes	Liposomal doxorubicin, Bevacizumab, and Everolimus [75]	mToR inhibitor (Everolimus) & PI3K inhibitor (Alpelisib) VEGFRi (Lucitanib) or Bevacizumab
ORR	NA	ORR = 28% with 4 complete responses and 7 partial responses	NA
DCR	NA	DCR = 40%	NA
OS/PFS	NA	NA	NA
DLT	NA	Grade 3 or higher adverse events included mainly Mucositis occurring in almost all patients, around 50% of infections, hand foot syndrome, and thrombocytopenia	NA
Patients characteristics	NA	Patients with advanced or metastatic TNBC who failed the standard therapy; meaning it did not improve their survival by at least three months, or they relapsed after standard therapy	NA
Price and feasibility	N/A	Doxorubicin Liposomal Trade name: Doxil^®^ Price: $387/10 mls https://www.drugs.com/price-guide/doxorubicin-liposomal + Bevacizumab Trade name: Avastin^®^ Price: $841/4mls https://www.drugs.com/price-guide/avastin + Everolimus Trade name: Afinitor^®^ Price: $4769/28 tablets https://www.drugs.com/price-guide/everolimus	Everolimus + Trade name: Afinitor^®^ Price: $4769/28 tablets https://www.drugs.com/price-guide/everolimus Alpelisib Trade name: Piqray^®^ Price: $24,859/56 tablets https://www.drugs.com/price-guide/piqray + Lucitanib Trade name: Tukysa^®^ Price: $13,789/60 tablets https://www.drugs.com/price-guide/tukysa OR Bevacizumab Trade name: Avastin^®^ Price: $841/4mls https://www.drugs.com/price-guide/avastin

*ORR* overall response rate, *DCR* disease control rate, *OS* overall survival, *PFS* progression free survival, *SD* stable disease, *PR* partial response, *CR* complete response, *DLT* disease limiting toxicity, *mTNBC* metastatic triple negative breast cancer

The main limitations of this, evidence-based treatment guide, include the fact that majority of the included clinical trials are only phase II clinical trials. This raises concerns about their generalizability to a broader population, as they often rely on variable and sometimes small sample sizes. Additionally, the inclusion criteria and studies designs vary across these trials, which can make comparisons between their results somewhat unreliable. One final limitation is the cost-effectiveness of regimens, as targeted therapies come with significantly high costs due to their early development stages (Table 9).

**Table 9:** A comparison between the guideline’s treatment, and the recommended evidence based guide for AR subtype

AR subtype	Guidelines’ treatment	Recommended evidence based guide	Recommended future clinical trials
Line of treatment	No standard treatments are available for the AR subtype	AR blocker (Enzalutamide)	AR blocker (Enzalutamide) + PIK3i (Alpelisib) or VEGFRi (Apatinib/Lucitanib)
		AR blocker (Enzalutamide) + PIK3i (Taselisib) (NCT01889238 & NCT02457910)	
ORR	NA	NA	NA
		NA	
DCR	NA	CBR = 33.3%	NA
		CBR = 35.7%	
OS/PFS	NA	OS = 17.6 months	NA
		PFS = 3.3 month	
		PFS = 3.4 month	
DLT	NA	Fatigue was the only grade 3 adverse event	NA
		The serious side effects was around 41.67% and they included febrile neutropenia, fever, acute appendicitis, benign neoplasms, and confusion	
Patient characteristics	NA	AR + ve TNBC patients	NA
		TNBC patients	
	NA	Enzalutamide Trade name: Xtandi^®^ Price: $15,571/120 capsules https://www.drugs.com/price-guide/tukysa + Taselisib NA Alpelisib can be used instead Alpelisib Trade name: Piqray^®^ Price: $24,859/56 tablets https://www.drugs.com/price-guide/piqray	Enzalutamide Trade name: Xtandi^®^ Price: $15,571/120 capsules https://www.drugs.com/price-guide/tukysa + Alpelisib Trade name: Piqray^®^ Price: $24,859/56 tablets https://www.drugs.com/price-guide/piqray OR Lucitanib Trade name: Tukysa^®^ Price: $13,789/60 tablets https://www.drugs.com/price-guide/tukysa

*ORR* overall response rate, *DCR* disease control rate, *OS* overall survival, *PFS* progression free survival, *SD* stable disease, *PR* partial response, *CR* complete response, *DLT* disease limiting toxicity, *mTNBC*; metastatic triple negative breast cancer

## Conclusions

In different phases of clinical trials, the protein targeted therapy provides a better efficiency and a much greater safety than the chemotherapeutics. Moreover, the overlapping nature of TNBC subtypes, previously proven by immunohistochemical research, was confirmed here by our analysis of different clinical trials’ phases, utilizing the given TNBC patient’s characteristics, outcomes, and applied regimens. This necessitates the genetic profiling consideration before initiating TNBC patients’ treatment in terms of BRCA mutations, RTK upregulation, mTOR upregulation, PD-1/PD-L1 upregulation, and AR positivity. We also recommended an evidence based treatment for TNBC patients that is advisable to be applied in future clinical trials. Our comprehensive review suggests that TNBC patients’ urgent need for standard treatment with minimum side effects and maximum efficiency to face the problems of current standard treatment. This can even turn cancer treatment into a safe chronic treatment. Hence, future clinical trials should consider maximizing the safety beside the efficacy of the targeted therapies aiming to validate their long-term use by cancer patients.

## Data Availability

The datasets used and/or analyzed in the current review are available in the clinical trials repository, ClinicalTrials.gov.
